# In Vivo Validation of a Novel Computational Approach to Assess Microcirculatory Resistance Based on a Single Angiographic View

**DOI:** 10.3390/jpm12111798

**Published:** 2022-10-31

**Authors:** Yongzhen Fan, Simone Fezzi, Pengcheng Sun, Nan Ding, Xiaohui Li, Xiaorong Hu, Shuang Wang, William Wijns, Zhibing Lu, Shengxian Tu

**Affiliations:** 1 Department of Cardiology, Zhongnan Hospital of Wuhan University, Wuhan 430071, China; 2 Institute of Myocardial Injury and Repair, Wuhan University, Wuhan 430072, China; 3 The Lambe Institute for Translational Medicine, the Smart Sensors Lab and Curam, University of Galway, University Road, H91 TK3 Galway, Ireland; 4 Division of Cardiology, Department of Medicine, University of Verona, 37129 Verona, Italy; 5 Shanghai Pulse Medical Technology Inc., Shanghai 200233, China; 6 Department of Cardiovascular Ultrasound, Zhongnan Hospital of Wuhan University, Wuhan 430071, China; 7 Biomedical Instrument Institute, School of Biomedical Engineering, Shanghai Jiao Tong University, Shanghai 200240, China

**Keywords:** coronary physiology, coronary pressure and flow, myocardial microcirculation, microvascular dysfunction, ischemia with non-obstructed coronary artery disease, angiography-derived physiology, functional coronary angiography, personalized invasive therapy in coronary artery disease

## Abstract

(1) Background: In spite of the undeniable clinical value of the index of microvascular resistance (IMR) in assessing the status of coronary microcirculation, its use globally remains very low. The aim of this study was to validate the novel single-view, pressure-wire- and adenosine-free angiographic microvascular resistance (AMR) index, having the invasive wire-based IMR as a reference standard. (2) Methods: one hundred and sixty-three patients (257 vessels) were investigated with pressure wire-based IMR. Microvascular dysfunction (CMD) was defined by IMR ≥ 25. AMR was independently computed from the diagnostic coronary angiography in a blinded fashion. (3) Results: AMR demonstrated a good correlation (r = 0.83, *p* < 0.001) and diagnostic performance (AUC 0.94; 95% CI: 0.91 to 0.97) compared with wire-based IMR. The best cutoff value for AMR in determining IMR ≥ 25 was 2.5 mmHg*s/cm. The overall diagnostic accuracy of AMR was 87.2% (95% CI: 83.0% to 91.3%), with a sensitivity of 93.5% (95% CI: 87.0% to 97.3%), a specificity of 82.7% (95% CI: 75.6% to 88.4%), a positive predictive value of 79.4% (95% CI: 71.2% to 86.1%) and a negative predictive value of 94.7% (95% CI: 89.3% to 97.8%). No difference in terms of CMD rate was described among different clinical presentations. (4) Conclusions: AMR derived solely from a single angiographic view is a feasible computational alternative to pressure wire-based IMR, with good diagnostic accuracy in assessing CMD.

## 1. Introduction

Coronary microvascular dysfunction (CMD) is a common under-diagnosed cause of myocardial ischemia, which yields an undeniable clinical and prognostic value both in acute (ACS) and chronic coronary syndromes (CCS) [1,2,3,4]. Several non-invasive physiological approaches to assess CMD have been developed, but their use in clinical practice has been very limited, due to technical and logistical drawbacks. The hyperemic microvascular resistance (HMR) and the index of microcirculatory resistance (IMR) are two invasive estimates of microvascular resistance, derived through a doppler- and a pressure thermistor-tipped coronary guidewire, respectively [5]. Microvascular resistance assessment has been shown to provide prognostic value in several clinical settings [6,7,8,9], whilst it allows the diagnosis of CMD in patients with anginal symptoms and no significant epicardial coronary disease [4,10]. Invasive IMR assessment in the cathlab is feasible with higher reproducibility than doppler-based indices and without the influence of physiological hemodynamic changes [11,12]. In spite of these positive features, the use of IMR in daily clinical practice is globally very low.

In order to facilitate the investigation of CMD, different angiography-based solutions have been proposed and validated against invasive IMR, based on computational flow analysis [13,14,15,16], and showing good accuracy in detecting CMD (~80%) [17]. All these indexes have been derived from quantitative flow ratio (QFR), an angiography-based index with good diagnostic metrics and prognostic value [18,19], whose computation is nevertheless hampered by a number of limitations [20]. In light of this, the single-view Murray’s law-based QFR (μQFR) has been recently developed and validated, showing excellent agreement with invasive fractional flow reserve (FFR). Its fast and reproducible computation reduces operator-dependance of the measurement [21].

The objective of this study was to develop and validate a novel method to assess microvascular resistance, based on μQFR algorithms and associated advantages. The angiography-derived microcirculatory resistance (AMR) is a wire-free and adenosine-free index, which aims at providing a valid alternative to invasive wire-based IMR assessment.

## 2. Materials and Methods

### 2.1. Study Design

The present study was a single-center observational study that included all the patients admitted to Zhongnan Hospital of Wuhan University between 2012 and 2020 who underwent invasive coronary microvascular assessment, according to clinical practice indication. Patients presenting with both ACS and CCS were included. ST-segment elevation myocardial infarction (STEMI) was defined as the occurrence of ongoing chest pain for at least 30 min associated with ST-segment elevation >2 mm in at least two contiguous leads or new left bundle branch block [22]. ACS also included non-STEMI or unstable angina with the occurrence of symptoms of myocardial ischemia and/or new significant ST-T changes on electrocardiogram and/or imaging evidence of new regional wall motion abnormalities, together with evidence of increased high-sensitivity troponin (above the 99th percentile of the upper reference limit) in the case of non-STEMI [23]. CCS was defined in the case of symptoms compatible with exertional myocardial ischemia and/or non-invasive evidence of coronary artery disease and/or inducible myocardial ischemia [3].

Overall, vessels with non-obstructive disease by angiographic visual estimation (i.e., area stenosis <50%) were included pre or post percutaneous coronary intervention (PCI). In the setting of STEMI undergoing primary PCI, invasive coronary physiology assessment of the culprit vessel was performed after flow restoration with thrombus aspiration and/or balloon dilatation and/or at completion of primary PCI. In patients with non-STEMI and unstable angina, invasive coronary physiology assessment was performed at completion of PCI of the culprit vessel. The identification of the culprit vessel was based on the combination of (1) angiographic appearance compatible with plaque instability or presence of thrombus, (2) electrocardiographic and echocardiographic findings. In the setting of CCS with obstructive coronary arteries, invasive coronary physiology assessment was performed on completion of PCI, whereas in the case of non-obstructive disease (angiographic area stenosis <50% and fractional flow reserve >0.8) it was performed during the diagnostic angiography.

Patients were enrolled on a prospective registry, and individual patient data were collected using standardized spreadsheets. Clinical exclusion criteria were: (1) left ventricular ejection fraction ≤50%; (2) estimated glomerular filtration rate <45 mL/min/1.73 m^2^); (3) severe coagulopathy or bleeding disorders; (4) allergy to iodine contrast agents or adenosine. Angiographic exclusion criteria included poor contrast opacification and severe vascular overlap or foreshortening of the interrogated vessel.

The retrospective study was approved by the Institutional Review Boards at Zhongnan Hospital of Wuhan University, Wuhan, China, which conforms to the declaration of Helsinki and Good Clinical Practice Guidelines of the China National Medical Products, National Medical Products Administration. All the patients provided their written consent for the anonymous collection of the data. A comprehensive study flow-chart is provided in Supplementary Figure S1.

### 2.2. μQFR and AMR Computation

A single-view μQFR analysis was computed, using the QFR software (AngioPlus Core, version V3, Shanghai Pulse Medical Technology Inc., Shanghai, China). The analysis was performed by an experienced and certified analyst at an independent academic core laboratory (CardHemo, Med-X Research Institute, Shanghai Jiao Tong University, Shanghai, China) who was blinded to invasive IMR data. The detailed methodology for single-view μQFR computation has been described previously [21]. In brief, after selecting the optimal angiographic view with minimal vessel overlap, the lumen contour of the interrogated coronary artery is automatically delineated, whilst the contrast flow velocity is derived from the length of the vessel centerline divided by the contrast filling time, and then converted into hyperemic flow velocity [24]. Subsequently, a frame with good contrast fill-in and full exposure of the lumen contour is selected as the analysis frame, and the lumen boundaries of both the interrogated vessel and major side branches are delineated automatically. The reference vessel diameter is then reconstructed, considering the step-down phenomenon across bifurcations based on the Murray bifurcation fractal law [20,25]. Finally, pressure drop is calculated, based on fluid dynamic equations with the above-mentioned hyperemic flow as the boundary condition [21]. Based on the pressure drop, the distal coronary pressure (P_d_) is obtained and μQFR computed as P_d_ divided by the mean aortic pressure (P_a_), while AMR is computed as P_d_ divided by the hyperemic flow velocity *Velocity_hyp_*.

$$\mathrm{AMR}=\frac{P_{d}}{Velocity_{hyp}}=\frac{P_{a}\times \mu QFR}{Velocity_{hyp}}$$

In this computation, an average aorta pressure of 86 mmHg during maximum hyperemia is assumed, as previously reported [26].

### 2.3. Wire-Derived IMR Measurement

Invasive physiology measurement was performed in a routine manner through a pressure thermistor-tipped coronary guidewire (St. Jude Medical, St. Paul, MN, USA) and thermodilution according to the standard procedures suggested by the RadiAnalyzer Xpress instrument (St. Jude Medical, St. Paul, MI, USA). In brief, the wire was calibrated, equalized and advanced towards the distal third of the coronary artery. One minute after intracoronary injection of 100 μg of isosorbide dinitrate, Pa, Pd and mean transit time (tTmean) were measured at baseline and during stable hyperemia induced by the intravenous infusion of adenosine (140 µg/kg/min). Thermodilution curves were obtained by the injection of 3 mL of room temperature 0.9% saline solution, and tTmean was calculated as the average of three transit time measurements during at least three separate injections.

IMR was then calculated as follows:

$$\mathrm{IMR}=P_{d}\;\left(\mathrm{hyperaemia}\right)\times tTmean\left(\mathrm{hyperemia}\right)$$

Pressure drift was checked and avoided by accurate pull-back of the pressure wire to the guiding catheter tip and defined as P_d_/P_a_ between 0.97 and 1.03. FFR was also recorded during this procedure.

### 2.4. Statistical Analysis

Continuous variables were recorded as mean ± standard deviation (SD) or median (25th percentile, 75th percentile), and categorical variables were recorded as count (percentage). Spearman’s correlation coefficient was used for the correlation analysis. Linear regression analysis was used to quantitatively define the relation between paired variables. Bland-Altman analysis was used for evaluating the analysis agreement between different analyzers. Using wire-derived IMR ≥ 25 as the reference standard [10], the area under the curve (AUC) by receiver-operating characteristic (ROC) curve analysis was used to evaluate the diagnostic performance of AMR. The Youden index was used to determine the best cutoff value of AMR in predicting IMR ≥ 25. The heterogeneity of sensitivity and specificity among different vessel types and clinical presentations was analyzed by I2 statistics. Inter-observer reproducibility of AMR computation was evaluated by two observers analyzing 30 vessels randomly selected from the study population in a blinded fashion. The paired measurements were compared using the correlation coefficient and paired sample *t* test. A *p*-value < 0.05 was considered significant, and all comparisons were two-sided. All statistical analyses were performed with MedCalc (version 20.1, MedCalc Software, Ostend, Belgium).

## 3. Results

### 3.1. Baseline Characteristics

Paired pressure wire-derived IMR and AMR were successfully acquired in 163 patients (257 vessels). Baseline demographics and vessel characteristics are reported in Table 1. The mean age was 62.8 ± 11.4 years. Almost half (*n* = 80; 49.1%) of the patients presented with acute MI, including STEMI (*n* = 44; 27.0%) and non-STEMI (*n* = 36; 22.1%). The remainder had unstable (*n* = 41; 25.2%), or stable angina, with obstructive (*n* = 28; 17.2%) or non-obstructive coronary arteries (*n* = 14; 8.6%). Among patients presenting with acute MI, 44 patients (27%) had MI ≤ 7 days prior to the index angiography, and among them 41 patients presented with STEMI and 3 presented with non-STEMI. The quantitative coronary analysis (QCA)-defined degree of diameter stenosis was 28.78 ± 9.24%, while median QFR value was 0.94 (0.91–0.97) and mean blood flow velocity 16.87 ± 5.42 cm/s (Table 2).

### 3.2. Correlation and Diagnostic Performance of AMR

**Figure 1:**
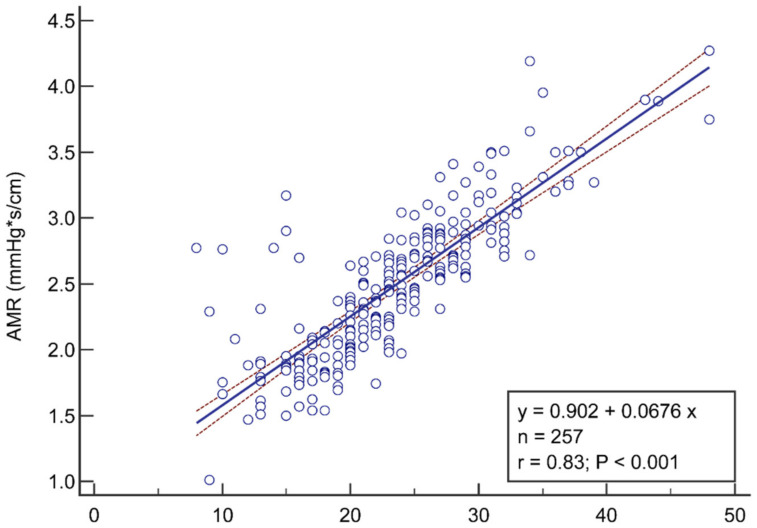
Correlation and the linear regression between AMR and IMR. AMR values (y axis) showed good correlation (r = 0.83, *p* < 0.001) with IMR (x axis). The red dotted line represents the 95% CI of the linear regression equation that was quantified as AMR = 0.90 + 0.07 × IMR. AMR, angio-derived microcirculatory resistance; IMR, index of microvascular resistance.

The mean values of pressure wire-based IMR was 23.6 ± 6.8 versus 2.5 ± 0.5 mmHg*s/cm for AMR (Table 2). AMR showed a good correlation (r = 0.83, *p* < 0.001) with IMR (Figure 1). The linear regression model indicated a quantitative linear relationship between AMR and IMR:

AMR = 0.90 + 0.07 × IMR

**Figure 2:**
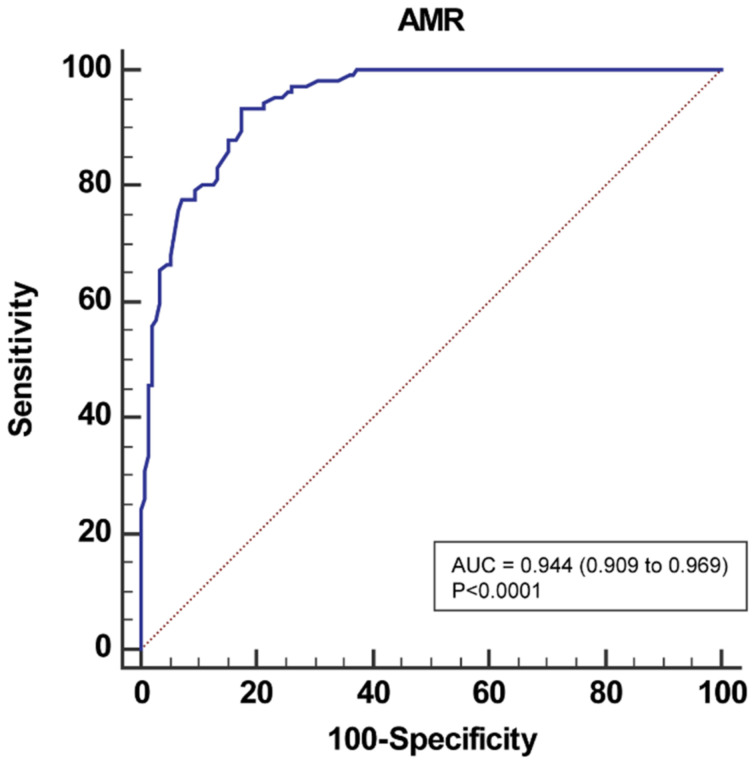
Receiver-operating characteristic curve analysis of AMR for identifying IMR ≥ 25U. AMR, angio-derived microcirculatory resistance; IMR, index of microvascular resistance.

At the ROC analysis (Figure 2), the area under the curve of AMR in predicting IMR ≥ 25 was 0.94 (95% CI: 0.91 to 0.97), with a sensitivity of 93.5% (87.0–97.3), a specificity of 82.7% (75.6–88.4), a positive predictive value of 79.4% (71.2–86.1) and a negative predictive value of 94.7% (89.3–97.8). Positive and negative likelihood ratios were 5.39 (3.80–7.70) and 0.08 (0.04–0.20), respectively, as shown in Table 3. AMR yielded an overall diagnostic accuracy of 87.2% (95% CI: 83.0% to 91.3%).

The best cutoff value for AMR in predicting IMR ≥ 25 defined by Youden test from the ROC analysis was 2.5 mmHg*s/cm.

### 3.3. The Influence of Vessel Type and Clinical Presentations on Sensitivity and Specificity of AMR

**Figure 3:**
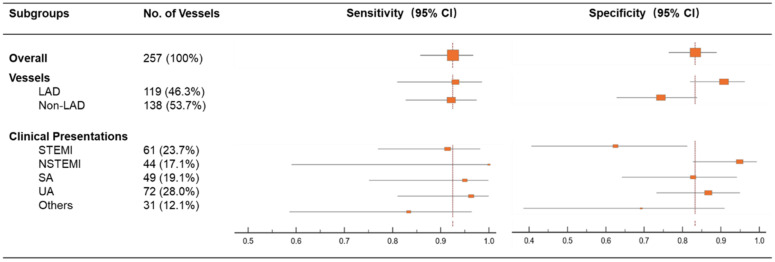
Sensitivity and specificity of AMR in different vessel types and clinical presentations. LAD, left anterior descending artery; NSTEMI, non-ST-segment elevation myocardial infarction; SA, stable angina; STEMI, ST-segment elevation myocardial infarction; UA, unstable angina.

The overall sensitivity and specificity of AMR > 2.5 mmHg*s/cm to predict IMR ≥ 25 was 93.5% and 82.7%. As shown in Figure 3, the sensitivity of AMR was not significantly influenced by the vessel type (e.g., left anterior descending vs other vessels). However, the specificity of AMR was significantly lower in patients with STEMI than with non-STEMI {62.5% (40.6–81.2) vs. 94.9% (82.7–99.4), *p* = 0.003}. Consistently, AMR in patients with MI ≤7 days had a comparable sensitivity {94.3% (80.8, 99.3) vs. 100% (59.0, 100.0), *p* = 0.746} but lower specificity {62.5% (40.6, 81.2) vs. 94.9% (82.7, 99.4), *p* = 0.003}.

No significant difference of MR values was observed between patients presenting with ACS or CCS, according to IMR (23.5 ± 7.0 vs 23.7 ± 6.2, *p* = 0.85) or AMR (2.5 ± 0.6 vs 2.6 ± 0.5, *p* = 0.10) assessment. The rates of CMD did not differ among the two populations (39.0% vs 47.5%, *p* = 0.20 for IMR; 47.5% vs 55.0%, *p* = 0.34 for AMR).

Similarly, no significant difference of MR values was observed between diabetic and non-diabetic patients, according to IMR (23.5 ± 7.1 vs 23.6 ± 6.6, *p* = 0.94) or AMR (2.4 ± 0.6 vs 2.5 ± 0.5, *p* = 0.42) assessment, with no significant difference in CMD rates. Individual MR scatter-plots according to clinical presentations are shown in Supplementary Figure S2.

### 3.4. Reproducibility of AMR Analysis

**Figure 4:**
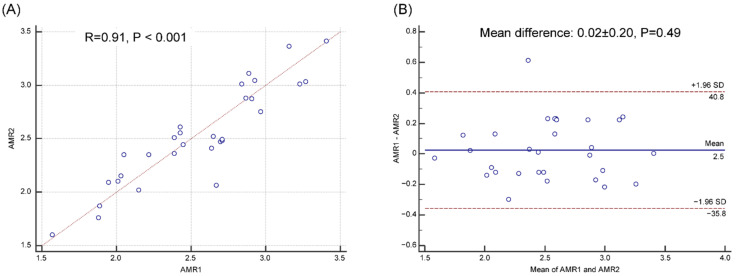
Reproducibility of AMR analysis. Correlation (**A**) and agreement (**B**) of AMR analyzed by two blinded investigators.

Inter-observer reproducibility depicted an excellent correlation (r = 0.91, *p* < 0.001) and absolute agreement at the Bland-Altman analysis (mean difference ± SD, 0.02 ± 0.20, *p* = 0.49), as shown in Figure 4.

## 4. Discussion

In this study we developed and validated a new pressure wire-free and adenosine-free index that investigates CMD, based on the analysis of a single angiographic view. The major findings of our study can be summarized as follows: (1) AMR showed good correlation (r = 0.83; *p* < 0.001) and diagnostic accuracy (87.2%; 95% CI: 83.0% to 91.3%) in predicting wire-based IMR; (2) AMR accuracy was not influenced by the investigated vessel, but a significantly lower specificity was observed during the first week after MI, and particularly in STEMI patients; (3) A low inter-observer variability was reported after repeated blinded AMR analysis.

Pressure wire-based MR can be investigated either by means of flow velocity, using a doppler transducer to derive the hyperemic microvascular resistance (HMR), or by means of transit time analysis, using thermodilution principles, to derive IMR. IMR and HMR are recommended to assess CMD in several clinical scenarios [5]. MR assessment has been shown to predict the risk of death and congestive heart failure after successful primary PCI in STEMI [6], and to properly stratify the risk of peri-procedural MI after elective PCI [7]. In the setting of CCS with and without obstructive coronary artery disease, IMR and HMR allow the diagnosis of CMD and stratify the risk in several specific patients subgroups, such as those with abnormal stress study or anginal symptoms without significant epicardial coronary disease (up to 70%), or those with residual angina after successful PCI (up to 30% of the cases) [4,10]. In the setting of heart transplantation, on top of its diagnostic and prognostic relevance, CMD assessment enables the tailoring of medical treatment, with reversibility of CMD after the implementation of the pharmacological treatment scheme [8,9,27]. Moreover, CMD was found to predict left ventricular dysfunction and a higher risk of adverse events in patients presenting with Takotsubo syndrome [28,29].

Although invasive IMR assessment is easier to obtain than HMR, its use in routine clinical practice is hampered by technical (i.e., the side effects of vasodilator agents required for stable hyperemia induction and complications related to vessel wiring) and economic issues, as well as by the operator’s reluctance to change, since decisions remain largely driven by angiography alone. This leads to an overall under-diagnosis of CMD, resulting in repeated hospitalizations, unnecessary coronary angiographies and adverse cardiovascular outcomes in the short and long term [4].

Therefore, several efforts have been made to enable CMD computation both on-line and off-line from angiography, with avoidance of the need to induce hyperemia and the use of dedicated pressure wires. This is expected to reduce procedural duration and costs, with the promise of broadening the adoption of CMD assessment in the real-world decision-making process.

According to our original analysis, AMR provides good correlation and diagnostic accuracy against invasive IMR, as the standard reference.

Of note, AMR computation overcomes two major drawbacks of the previously proposed angiography-derived IMR indexes. On the one hand, AMR computation, similar to the doppler-based HMR, is based on the estimated hyperemic flow velocity instead of the mean transit time [14,15,16]. The use of mean contrast transit time, required for the thermodilution-based IMR, is strictly dependent on the length of the region of interest, leading to potential pitfalls and operator-dependent variability. Indeed, the longer the segment of interest, the higher the mean contrast transit time and thus the higher the angiography-derived IMR. AMR computation based on the estimated hyperemic flow velocity showed high inter-observer reproducibility and reduced computational time (less than one minute). It should be acknowledged that HMR is theoretically a more robust surrogate of MR compared with IMR; indeed, doppler-based flow velocity is widely recognized to yield a more accurate estimate of flow velocities compared with transit time, especially at slower flow rates (i.e., resting flow). This might affect the accuracy of angiography-derived IMR, when such computation is performed through mean transit time under resting conditions [30,31]. Moreover HMR correlates better with several noninvasive and invasive measures of perfusion [32].

On the other hand, previously reported angiography-derived IMR indexes were based on QFR computation from two angiographic projections, which is not devoid of several limitations. Firstly, it requires two angiographic views with at least 25° separation from each other, and with minimal vessel foreshortening and overlap. Secondly, a linear tapering of the reference vessel size is assumed during QFR computation, with reduced accuracy in specific settings (i.e., bifurcation lesions). Conversely, the novel μQFR, besides being derived from a single angiographic projection, provides an artificial intelligence-empowered delineation of vessel contours, frame counting, and a more accurate reconstruction of reference vessel size, with a fast and reproducible computation, all of which reduce operator-dependence of the measurement [21]. Interestingly, in our study, retrospective analysis of both μQFR and AMR was feasible in the vast majority of the cases (~85% as provided in Supplementary Figure S1), whilst two-view QFR analysis was reported to have lower feasibility in retrospective analysis, with half of the cases not analyzable, due to the lack of a second appropriate angiographic projection [33,34].

The accuracy of AMR computation was shown to be independent from the analyzed vessel segment, while a significant impact of the clinical presentation was observed. In patients with STEMI and those presenting ≤7 days after acute MI, AMR showed a significantly lower specificity, compared with NSTEMI, MI >7 days or CCS. Similar results were previously reported, suggesting that the agreement between angiography-derived IMR and invasive IMR was very close in NSTEMI and CCS, whereas it was less accurate in the STEMI setting. The inherently higher biological variability of IMR in a STEMI setting could explain such a difference, as the absolute numerical IMR values were less well correlated with the standard of reference in the case of extreme CMD [13,14,35]. This might be related to the hyper-sympathetic activation and the profound structural microvascular deterioration since the early phases (i.e., microvascular clotting, leukocyte adhesion and distal embolization) [13,14,35]. Thus, in the setting of functional CMD, the use of hyperemic angiograms to compute AMR might be considered, as previously reported for the FFR/QFR disagreement [36]. Interestingly, AngioPlus Core QFR software allows the computation of AMR both in resting and hyperemic conditions. In the present analysis, hyperemic AMR was derived through algorithms, to convert the resting flow velocity into hyperemic flow velocity, the same being used in the computation of QFR based on resting angiography [26]. When hyperemic angiograms are available, the software is settled to directly calculate hyperemic flow velocity and hyperemic AMR, without applying the algorithmic interpretation.

To the best of our knowledge, this is the first study reporting a temporal influence on the accuracy of angiography-derived MR in assessing CMD in the setting of MI. It should be noted that both Tebaldi et al. and Mejia-Renteria et al. included only stable CCS in their analyses, whereas Scarsini et al. included both ACS and CCS. They provided a non-hyperemic angiography-derived IMR that did not take into account the estimated hyperemic velocity in its computation [13,15,16]. Therefore, further investigations are warranted before conclusive inferences can be drawn.

**Figure 5:**
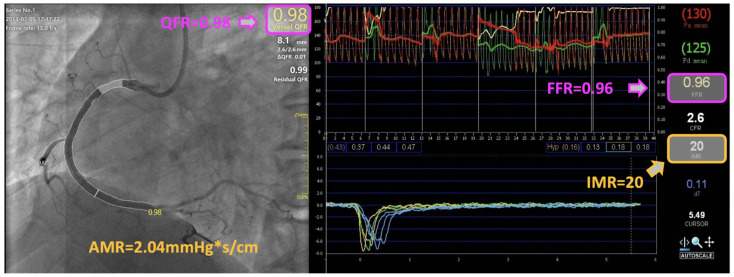
Derivation of coronary microvascular resistance from coronary angiography. An illustrative case-example of integrated angiography-derived epicardial and microvascular physiology assessment in a patient presenting with unstable angina: values of the pressure-wire thermodilution-derived index of microvascular resistance (IMR) and angio-derived microcirculatory resistance (AMR) index showed concordance and good agreement. AMR, angio-derived microcirculatory resistance; FFR, fractional flow reserve; IMR, index of microvascular resistance; QFR, quantitative flow ratio.

To conclude, AMR is a novel angiography-derived index that aims at increasing the availability of a prompt and integrated assessment of the microvascular function. This might represent a major step forward towards precision medicine implementation, with improvement in the diagnostic and therapeutic pathway of all the patients referred for invasive angiography assessment, over a wide span of clinical settings. Interestingly, according to our analysis, this approach led to the detection of a high CMD rate, even in patients with CCS in whom high MR was not anticipated from clinical variables. Therefore, the use of angiography-based computational technologies offers the net advantage of a systematic and comprehensive physiological assessment of both epicardial and microvascular determinants of the coronary circulation, in a fast, simple, and reproducible manner. A comprehensive illustration of the integrated coronary physiological computation based on single-view coronary angiography is provided in Figure 5.

## 5. Study Limitations

There are some limitations that should be considered.

Firstly, even if patient demographics and coronary angiograms were collected prospectively, we performed post hoc AMR assessment. However, no selection bias was introduced, since AMR computation was attempted in all available cases meeting the study inclusion criteria.

Secondly, the study sample size might not allow the identification of all variables that can potentially influence AMR computation. To the best of our knowledge though, this study, including more than 250 vessels, is one of the largest available validation studies on angiography-derived MR computation methods [14,15,16].

The population included in the original study presented with high average QFR values and a low degree of stenosis established by quantitative coronary analysis, with many vessels devoid of significant obstruction, expanding the scope of CMD assessment by angiography-derived methods. Notably, Meja-Renteria et al. validated their angiography-derived IMR index in vessels with significant coronary artery disease (mean QFR value 0.74 vs. 0.94 in the present study) [16]. The assessment of AMR in vessels with more severe obstructive coronary disease deserves further investigation. This also applies to patients presenting with STEMI, given the observed lower specificity and positive predictive value.

In this study cohort, AMR was validated against thermodilution-based IMR, while no direct comparison with the doppler-based HMR was available. Future dedicated studies are warranted.

Lastly but importantly, the impact of AMR values on clinical outcomes in different patient subgroups deserves further investigation in prospective trials, with adequate follow-up.

## 6. Conclusions

Microvascular resistance can be properly derived from coronary angiography, with no need for dedicated pressure wires and hyperemic agents. In this validation study, AMR yielded a clinically useful degree of diagnostic accuracy in detecting CMD in different settings, versus wire-based IMR as a reference standard. AMR provides a valid, reproducible, widely available and fast computational alternative to wire-based IMR measurement during the routine angiographic evaluation of patients with suspected or known coronary artery disease.

## Figures and Tables

**Table 1:** Baseline demographic characteristics (*n* = 163 patients).

Patient Characteristics	*n* = 163
Age, years	62.8 ± 11.4
Male	105 (64.4%)
LVEF%	58.0 ± 7.6
Hypertension	107 (65.6%)
Hyperlipidemia	58 (35.6%)
Diabetes mellitus	41 (25.2%)
Smoking history	58 (35.6%)
Previous PCI	10 (6.1%)
Previous CABG	0 (0.0%)
Clinical presentation	*n* = 163
ACS	121 (74.2%)
Myocardial infarction	80 (49.1%)
STEMI	44 (27.0%)
MI ≤ 7d	41 (25.2%)
MI > 7d	3 (1.8%)
NSTEMI	36 (22.1%)
MI ≤ 7d	3 (1.8%)
MI > 7d	33 (20.3%)
Unstable angina	41 (25.2%)
CCS	42 (25.8%)
Obstructive CAD	28 (17.2%)
Non-obstructive CAD	14 (8.6%)

CABG, coronary artery bypass grafting; CAD, coronary artery disease; LVEF, left ventricle ejection fraction; MI, myocardial infarction; NSTEMI, non-ST-segment elevation myocardial infarction; PCI, percutaneous coronary intervention; STEMI, ST-segment elevation myocardial infarction.

**Table 2:** Vessel characteristics (*n* = 257).

Vessel	
LAD	119 (46.3%)
LCX	60 (23.3%)
RCA	78 (30.3%)
FFR	0.91 (0.87, 0.96)
QFR	0.94 (0.91, 0.97)
Flow velocity, cm/s	16.87 ± 5.42
IMR	23.6 ± 6.8
AMR, mmHg*s/cm	2.5 ± 0.5
Reference vessel diameter, mm	2.63 ± 0.68
MLD, mm	1.87 ± 0.55
DS, %	28.78 ± 9.24
Length, mm	14.53 ± 9.08

AMR, angio-derived microcirculatory resistance; DS, degree of stenosis; FFR, fractional flow reserve; IMR, index of microvascular resistance; LAD, left anterior descending; LCX, left circumflex artery; MLD, minimal lumen diameter; QFR, quantitative flow ratio; RCA, right coronary artery.

**Table 3:** The diagnostic performance of AMR in predicting IMR ≥ 25.

Best Cutoff Value for AMR	AMR > 2.5
Accuracy, % (95% CI)	87.2 (83.0–91.3)
Sensitivity, % (95% CI)	93.5 (87.0–97.3)
Specificity, % (95% CI)	82.7 (75.6–88.4)
PPV, % (95% CI)	79.4 (71.2–86.1)
NPV, % (95% CI)	94.7 (89.3–97.8)
+LR, (95% CI)	5.39 (3.80–7.70)
−LR, (95% CI)	0.08 (0.04–0.20)

LR, likelihood ratio; NPV, negative predictive value; PPV, positive predictive value.

## Data Availability

The data that support the findings of this study are available from the corresponding author upon reasonable request.

## Supplementary Materials

The following supporting information can be downloaded at: https://www.mdpi.com/article/10.3390/jpm12111798/s1, Figure S1: Study flow-chart. Figure S2: Scatter plot of microvascular resistance according to the clinical presentation and the presence of diabetes mellitus.
