# Supplementary material for: In Vivo Validation of a Novel Computational Approach to Assess Microcirculatory Resistance Based on a Single Angiographic View
Source: J Pers Med. 2022 Oct 31;12(11):1798. doi: 10.3390/jpm12111798 (PMC9692562; doi:10.3390/jpm12111798)
Supplement: Supplementary file 1 [file jpm-12-01798-s001.zip › jpm-1882470-supplementary.pdf]

[Supplementary Material]

In vivo validation of a novel computational approach to assess microcirculatory resistance based on a single angiographic view

Yongzhen Fan [1,2], Simone Fezzi [3,4], Pengcheng Sun [5], Nan Ding [5], Xiaohui Li [5], Xiaorong Hu [1,2], Shuang Wang [6], William Wijns [3], Zhibing Lu [1,2,*] and Shengxian Tu [7,*]

1 Department of Cardiology, Zhongnan Hospital of Wuhan University, Wuhan 430071, China
2 Institute of Myocardial Injury and Repair, Wuhan University, Wuhan 430072, China
3 The Lambe Institute for Translational Medicine, the Smart Sensors Lab and Curam, University of Galway, University Road, H91 TK3 Galway, Ireland
4 Division of Cardiology, Department of Medicine, University of Verona, 37129 Verona, Italy
5 Shanghai Pulse Medical Technology Inc., Shanghai 200233, China
6 Department of Cardiovascular Ultrasound, Zhongnan Hospital of Wuhan University, Wuhan 430071, China
7 Biomedical Instrument Institute, School of Biomedical Engineering, Shanghai Jiao Tong University, Shanghai 200240, China

Supplementary Figure S1. Study flow-chart

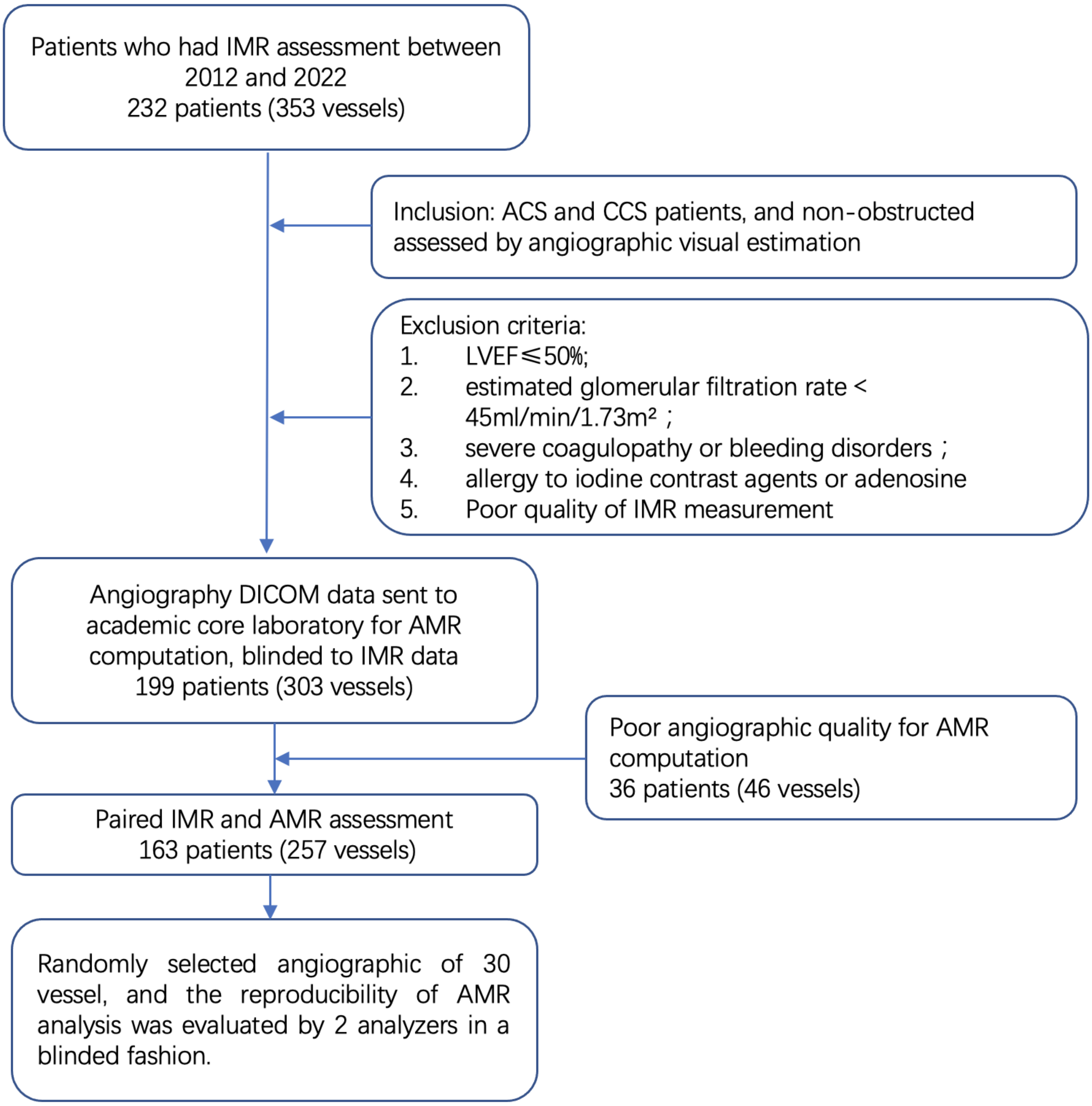

AMR, angio-derived microcirculatory resistance; IMR, index of microvascular resistance; LVEF, left ventricle ejection fraction

Supplementary Figure S2. Scatter plot of microvascular resistance according to the clinical presentation and the presence of diabetes mellitus

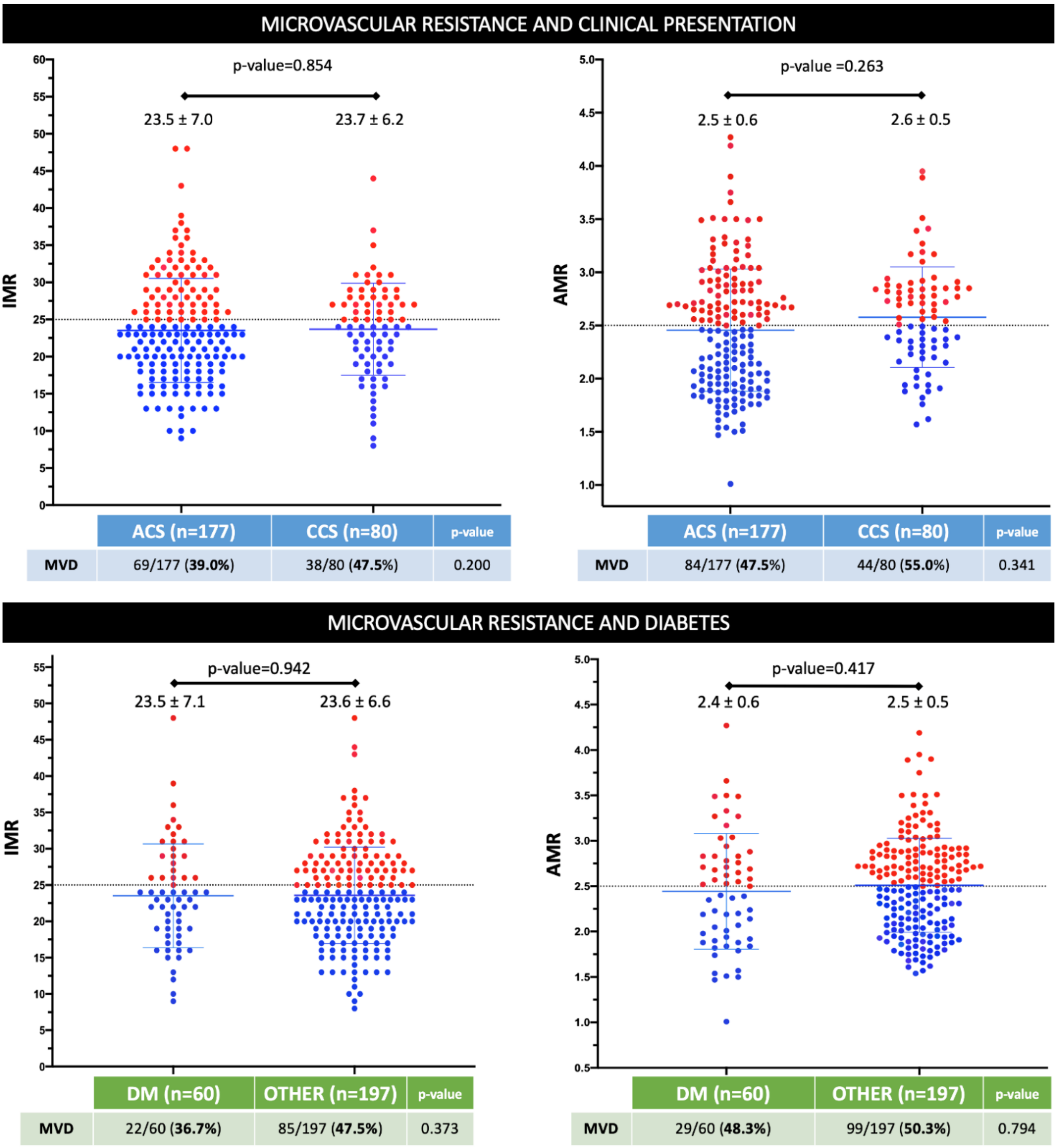

ACS, acute coronary syndrome; AMR, angio-derived microcirculatory resistance; CCS, chronic coronary syndrome; DM, diabetes mellitus; IMR, index of microvascular resistance; MVD, microvascular disfunction
